# Differentiated Thyroid Cancer with Extrathyroidal Extension: Prognosis and the Role of External Beam Radiotherapy

**DOI:** 10.4061/2010/183461

**Published:** 2010-05-06

**Authors:** Michael A. Sia, Richard W. Tsang, Tony Panzarella, James D. Brierley

**Affiliations:** ^1^Department of Radiation Oncology, Tom Baker Cancer Centre, Calgary, AB, Canada T2N 4N2; ^2^Department of Radiation Oncology, University of Calgary, Calgary, AB, Canada T2N 4N1; ^3^Department of Radiation Oncology, Princess Margaret Hospital, Toronto, ON, Canada M5G 2M9; ^4^Department of Radiation Oncology, University of Toronto, Toronto, ON, Canada M5S 3E2; ^5^Department of Biostatistics, Princess Margaret Hospital, University of Toronto, Toronto, ON, Canada M5S 2M9; ^6^Dalla Lana School of Public Health, University of Toronto, Toronto, ON, Canada M5T 3M7

## Abstract

A study was performed to identify variables that affected cause-specific survival (CSS) and local relapse-free rate (LRFR) in patients with differentiated thyroid cancer (DTC) and extrathyroid extension (ETE) and to examine the role of external beam radiotherapy (XRT). Prognostic factors were similar to those found in studies of all patients with DTC. In patients with postoperative gross residual disease treated with radiotherapy, 10-year CSS and LRFR were 48% and 90%. For patients with no residual or microscopic disease, 10-year CSS and LRFR were 92% and 93%. In patients older than 60 years with T3 ETE but no gross residual disease postoperatively there was an improved LRFR at 5 years of 96%, compared to 87.5% without XRT (*P* = .02). Patients with gross ETE benefit from XRT and there may be a potential benefit in reducing locoregional failure in patients over 60 years with minimal extrathyroidal extension (T3).

## 1. Introduction

The role of radiotherapy in the management of differentiated thyroid cancer remains controversial with the majority of patients treated with thyroidectomy and radioactive iodine alone [1]. However, there is a subset of patients who are at high risk for local recurrence who may benefit from adjuvant external radiation therapy. The difficulty in determining the optimal management of patients is the lack of randomized control trials and the reliance on single institution prospective databases and retrospective reviews, complicated by changes in clinical practice, pathology assessment, and surgical techniques over time. Despite this, the British Thyroid Association recommends XRT in patients over 60 years of age with extensive extranodal spread after optimal surgery, even in the absence of evident residual disease [2], and the American Thyroid Association guidelines have recommended that “The use of external beam irradiation should be considered in patients over age of 45 with grossly visible extrathyroidal extension (T4a and T4b) at the time of surgery and a high likelihood of microscopic residual disease” [3]. 

Several factors have been identified to be prognostic in determining survival. These factors include age, tumor size, grade, presence of macroscopic disease after surgical resection, and presence of distant metastases [4, 5]. Additionally extrathyroidal extension (ETE) has been identified as carrying a higher risk for local recurrence [4, 6, 7]. The definition of the AJCC and UICC T Category for thyroid cancer changed in 2002 and reflects the importance of varying degrees of ETE [8, 9]. There are three different levels of extent. T3 represents minimal ETE to sternothyroid or perithyroid tissue or any tumor greater than 4 cm. T4a includes invasion into subcutaneous soft tissue, larynx, trachea, esophagus, and recurrent laryngeal nerve. T4b includes invasion into prevertebral fascia, mediastinal vessels, or encasement of the carotid artery. In a retrospective review of 1067 patients with ETE treated by surgery alone, Ito et al. reported that minimal ETE (T3) did not have a deleterious effect on relapse-free survival (RFS) compared to no extrathyroidal extension, but that massive ETE (T4) did [10]. 

An analysis of a retrospective study from Princess Margaret Hospital (PMH) has previously shown that patients over the age of 60 with ETE and no gross residual disease after surgery had significantly improved 10-year cause-specific survival (CSS) (81.0% versus 64.6%, *P* = .04) and 10-year local relapse-free rates (LRFRs) (86.4% versus 65.7%, *P* = .04) following adjuvant external radiation therapy [11]. Patients under 60 had improved local control, but not survival. The indications for radiotherapy at PMH have changed and have been refined over the years. Currently, the indications for XRT are patients over the age of 45 years with T4a or T4b tumors, or gross residual disease following thyroidectomy. Previously XRT was considered in all patients with ETE, but radiotherapy is now not advised for patients with T3 tumors (minimal ETE). This study is a retrospective review performed to further assess the role of XRT in patients with ETE and identify prognostic factors for local relapse-free rates and cause-specific survival in patients with differentiated thyroid cancer and ETE that might help more clearly to define who are at high risk and may benefit from XRT and to test the hypotheses that young patients with T4b disease benefited from XRT while older patients with T3 disease did not.

## 2. Materials and Methods

Following local ethics review board approval, a retrospective chart review of 323 patients with differentiated thyroid carcinoma (papillary or follicular) and known ETE, referred for adjuvant management and registered between January 1958 and December 1999, was conducted. The identification of 369 potentially eligible patients was previously described by Brierley et al. [11]. The pathology slides of all patients were reviewed at PMH on referral. As in our previous study, because of changes in the distribution of patients with follicular as opposed to papillary thyroid cancer, presumably due to changes in pathologists interpretation, patients with papillary or follicular tumors were analyzed together [11]. Of the 369 patients, nine were not considered due to difficulty in obtaining the source documentation for this chart review, and a total of 37 patients were excluded because they were referred for recurrent disease, for followup with primary treatment elsewhere, and for second opinion, or they had distant metastatic disease at presentation.

The sample size was not based on prestudy considerations of statistical power but on the available number of patients within the relevant time period. Cause-specific survival (CSS) and local relapse-free rates (LRFRs) were calculated by the cumulative incidence method. LRFR was defined as freedom from relapse in the neck, either thyroid bed or cervical lymph nodes, based on clinical examination or diagnostic imaging. Using the cumulative incidence method, competing risks were not censored, but acknowledged in the calculation. For CSS, the competing event was death from among other causes. For LRFR, competing events included deaths or metastatic failures

Univariate log-rank testing and multivariate Cox proportional hazards regression modeling analysis were performed on prognostic variables; this included the effect of treatment. Variables examined were histology, gender, age, tumor size, T category, differentiation, nodal involvement, postoperative status, multifocality, margin status, and lymphovascular involvement. In accordance with previous analyses from our institution, age was analyzed as a continuous variable and on a categorical basis (<45, 45–60, >60) [5, 11, 12]. Tumor size was recorded and analyzed as a categorical variable, not a discrete variable; as in previous analyses, the categorical variables were <1 cm, ≥1 cm and <4 cm, and ≥4 cm as per earlier editions of TNM. Extent of ETE was categorized according to TNM 6th edition [8, 9]. Patients with metastatic disease on first posttherapy radioactive iodine scan were considered to have metastases at time of presentation. Followup data was obtained through a review of patient charts and electronic records. For patients that were not routinely followed at PMH, questionnaires were sent to attending physicians, followup phone calls were made to attending physicians, and cancer registry data was reviewed. 

As a result of multiple significance testing (from the prognostic factor analysis and several subgroup analyses, on two endpoints) the Type I error is inflated so it is plausible that some of the effects presented as statistically significant are spurious. However, as it has been argued that the adjustment of *P*-values with the Bonferroni method creates more problems than it solves [13], the *P*-values in this report are unadjusted for multiple comparisons. Moreover, given the limited sample size, our subgroup analyses have low power (i.e., an inflated Type II error) to detect statistically significant differences. Competing risk failure probabilities and plots were produced with the cmprsk package using version 2.6.0 in R. All other analyses were performed using SAS version 9.1.

## 3. Results

### 3.1. Patient Characteristics

**Figure 1:**
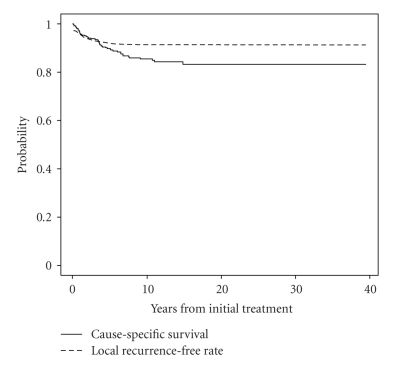
Cause-specific survival and local recurrence-free rate for patients with differentiated thyroid cancer and extrathyroidal extension. The overall 5- and 10-year cause-specific survivals are 89.8% (SE 0.03%) and 85.4% (SE 0.04%), respectively. The overall 5- and 10-year local relapse-free rates are 92.5% (SE 0.02%) and 91.7% (SE 0.02%), respectively.

The distribution of patient characteristics, candidate prognostic factors and treatments, are summarized in Table 1. The median followup among living cases (*n* = 235) was 10.8 years with a range from 0.6 to 39.5 years. The overall 5- and 10-year local relapsefree rates are 92.5% (SE 0.02%) and 91.7% (SE 0.02%), respectively. Of 323 patients, 56 have failed locally or regionally. The overall 5- and 10-year cause-specific survival rates are 89.8% (SE 0.03%) and 85.4% (SE 0.04%), respectively (Figure 1). Of 323 patients, 88 died; 46 died of disease and 37 died of other causes. In 5 cases, the cause of death was unclear. Of patients who had data regarding site of disease at time of death, 71% had distant metastasis, while 29% had local regional disease alone.

### 3.2. Prognostic Factor and Effects of Treatment Analyses: Baseline Variables

 The candidate prognostic factors and effects of treatment on cause-specific survival and local relapse-free rates were subjected to univariate and multivariate analyses. Local invasion of subsites (i.e., larynx, trachea, esophagus, prevertebral fascia, mediastinal vessels, etc.) for T4a and T4b was collected but not included in the analysis as the number of events for each subsite was small.

 Factors found to be significant for a worse cause-specific survival on univariate analysis are listed in Table 2. Unexpectedly patients with multifocal disease had better cause-specific survival. With respect to treatment, no difference in cause-specific survival was observed in patients receiving radioactive iodine ablation. Patients who underwent near total thyroidectomy had better outcomes. The factors which carried a statistically significant association with outcome on univariate analysis for local relapse rates are listed in Table 2. T category was not a statistically significant prognostic factor for local relapse-free rates. Treatment modalities (radioactive iodine ablation, XRT, and surgery) did not influence overall local relapse-free rates. However, surgery was of borderline significance.

 Multivariate analysis was performed on the majority of candidate prognostic factors found to be significant on univariate testing (Table 3). The factors statistically significant for improved cause-specific survival were postoperative status (no residual), papillary histology, and younger age—<45. The presence of multifocal disease remained a significant favorable prognostic factor for cause-specific survival. Smaller tumor size (<1 cm and 1–4 cm) and postoperative status (no residual) were significant for improved local relapse-free rates. There was a trend towards significance with age (*P* = .09). There was no effect from T (T3, T4a, or T4b) category for CSS or LRFR on the multivariate model. No association was seen between treatment modalities and improved cause-specific survival or local relapse-free rates.

### 3.3. Subgroup Analyses

**Figure 2:**
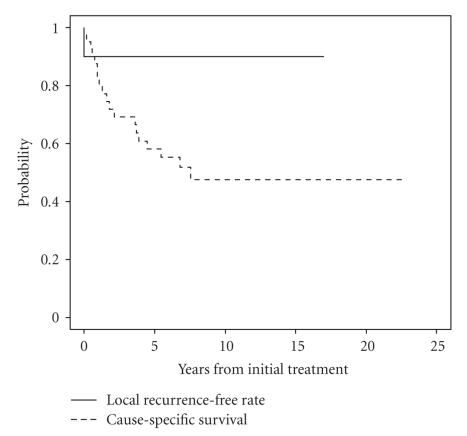
Cause-specific survival and local recurrence-free rate for patients with gross residual disease treated with RT; 10-year CSS and LRFR were 48% and 90%, respectively.

**Figure 3:**
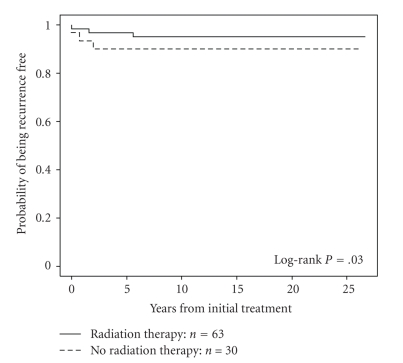
Local recurrence-free rate for patients over 45 years of age with T3 tumors treated with and without RT, 96.8% versus 90% at 5 years, respectively, *P* = .03.

**Figure 4:**
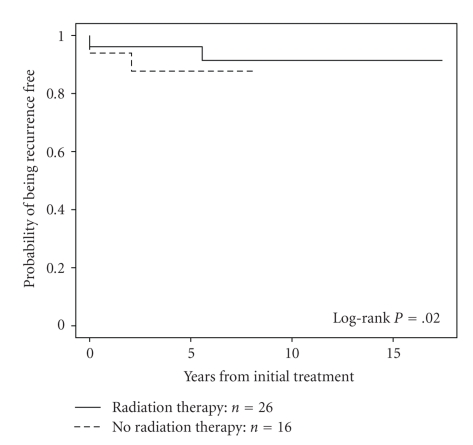
Local recurrence free rate for patients over 60 years of age with T3 tumors treated with and without RT, 96% versus 87.5%, at 5 years, respectively, *P* = .02.

In 40 patients with postoperative gross residual disease treated with radiotherapy, 10-year CSS and LRFR were 48% and 90%, respectively (Figure 2). Further subgroup analyses were performed in patients without gross residual disease after surgery, that is, no residual disease or microscopic residual. Patients were subdivided based on age (less than 45 years, 45–60 years, and greater than 60 years) and extent of extrathyroidal extension (T3, T4a, and T4b). Patients were also analyzed based on a combination of age and T category. XRT did not result in any significant difference in CSS for any subgroup. There was a trend in favor of improved LRFR in T3 tumors with XRT (94.8% versus 87.3% at 5 years, *P* = .08). There was a significant benefit in patients over 45 with T3 disease treated with XRT at 5 years. The LRFRs were 96.8% versus 90%, *P* = .03, (Figure 3), although in this subgroup there was a trend towards more use of RAI in patients who also had XRT. There was however no significant difference in LRFR for patients between 45 and 60, with the benefit being in those over 60 (96% versus 87.5%, *P* = .02, Figure 4), and in this subgroup there was no difference in the use of RAI although the number of patients was small. There was no benefit with XRT in T3 patients less than 45. Also there was no apparent benefit to XRT for local relapse-free rates in T4 tumors in those less than 45 years or greater than 60 years, but the numbers were relatively small.

## 4. Discussion

This study demonstrates a potential benefit to radiotherapy in reducing locoregional failure in patients over 60 years with minimal extrathyroidal extension (T3). The role of XRT in differentiated thyroid cancer remains unclear due to the lack of prospective randomized studies and the reliance on evidence from retrospective studies. The use of XRT in the management of thyroid cancer varies among centers, but several retrospective studies have shown a benefit with radiotherapy [14–22] and it is now recommended by several treatment guidelines in certain high-risk groups [2, 3, 23, 24]. Our current indications for adjuvant radiotherapy include patients over the age of 45 years with gross residual disease or over 45 years with clinical gross extrathyroidal invasion (T4), but not for patients with T3 tumors (minimal ETE). 

Age has been shown to be a significant factor for determining prognosis in differentiated thyroid cancer that is the only tumor type in which the AJCC and UICC use age for stage determination. Our previous study in [11] and another in [25] showed a greater worsening of outcome for patients over 60 years of age. Extrathyroidal extension has also been identified as a prognostic factor carrying a higher risk for local recurrence [5–7]. Following our recent retrospective analysis of adjuvant radiotherapy although no benefit to adjuvant radiotherapy was seen in patients less than 60 years with ETE, we speculated that, since the definition of ETE encompasses a large range of disease extent from minimal invasion of perithyroidal fat to invasion of the prevertebral fascia, there might still be a benefit to adjuvant radiotherapy in carefully selected high-risk patients under 60 years. Conversely, we also speculated that there might be a subset of patients over 60 years with minimal ETE that did not benefit from adjuvant radiotherapy. 

We were unable to demonstrate a benefit with radiotherapy for cause-specific survival and local relapse-free rates in T4 tumors in those less than 45 years, but the number of events was small. Also no benefit was seen with radiotherapy in those over 60 years with T4 disease and also the number of events was small. There was a significant benefit for local relapse-free rates, but not for cause-specific survival in patients over 60 years with T3 disease treated with radiotherapy. This benefit in patients with T3 disease was unexpected especially in light of the findings of Ito et al. that minimal ETE did not carry a poor prognosis for RFS for patients over the age of 45 [10], although they did not report whether there was a difference in RFS in T3 tumors by age greater or less than 60. It could be speculated that for patients greater than 60 there is a difference in outcome for patients with limited ETE (seen only microscopically), compared with those tumors with invasion into sternothyroid or perithyroid tissues noted at the time of surgery. The potential benefit of XRT if it really exists is small in T3 disease and the data should not be overinterpreted given that this is a retrospective review of patients seen over a long-time period with changing practices in surgery, radiotherapy, and the use of iodine. Another caveat is that multiple statistical comparisons were explored in subgroups, which increased the chances of finding a significant difference by chance alone.

The benefit of radiotherapy in differentiated thyroid cancers with gross residual disease has been shown in several studies [14, 15, 17, 18]. Chow and colleagues [26] demonstrated that extent of postsurgical residual disease was associated with cause-specific survival and that, when this factor was included in multivariate analysis, it overrode the other prognostic factors (size of tumor, extrathyroidal extension, lymph node metastases, and type of surgery). Analysis from our institution has also found postoperative presence of macroscopic residual disease to be a significant factor for 10-year local relapse-free rates and cause-specific survival [5]. In this study, patients with postoperative gross residual disease treated with radiotherapy had 10-year cause-specific survival and local relapse-free rates of 48% and 90%, respectively, demonstrating that even if disease is controlled in the neck there is a high risk of death from distant metastatic disease.

## 5. Conclusions

Prognostic factors for patients with ETE are similar to those of any patient with differentiated thyroid cancer. Although radiotherapy is of benefit in patients with gross residual disease, we were unable to define a subset of patients less than 45 years who benefited from adjuvant radiotherapy. There may be a potential benefit to radiotherapy in reducing locoregional failure in patients over 60 years with minimal extrathyroidal extension (T3). There remains uncertainty in how to select high-risk patients for radiotherapy. The potential benefit needs to be balanced against the generally excellent outcomes with surgery and radioactive iodine as well as the expected side effects of radiotherapy, although these may be reduced with modern radiation techniques [27].

## Figures and Tables

**Table 1:** Distribution of candidate prognostic factors and treatments.

	All *N* = 323	
	*N*	%
Histology		
Follicular	60	18.58
Papillary	261	80.80
Gender		
Female	239	73.99
Male	84	26.01
Age		
<45	134	41.49
45–60	95	29.41
>60	93	28.79
Size		
<1 cm	20	6.19
1–4 cm	187	57.89
>4 cm	72	22.29
Differentiation		
Moderate	37	11.46
Poor	52	16.10
Well	98	30.34
Nodal involvement		
No	148	45.82
Yes	149	46.13
Postoperative status		
Gross	28	8.67
No residual/microscopic	259	80.19
Multifocal		
No	187	57.89
Yes	119	36.84
Margin status		
Negative	195	60.37
Positive	92	28.48
LVI (Lymph vascular Invasion)		
No	87	26.93
Yes	130	40.25
T category		
3	169	52.32
4a	91	28.17
4b	58	17.96
Surgery		
Biopsy	22	6.81
Lobectomy	64	19.81
Near total	80	24.77
Radical	7	2.17
Total	146	45.20
RAI (Radioactive Iodine)		
No	65	20.12
Yes	258	79.88
XRT		
No	115	35.60
Yes	208	64.40

**Table 2:** Univariate analysis and Log-rank *P* value: summary.

Variable	CSS	LRFR
Histology (*N* = 321)	<0.0001	0.15
Gender (*N* = 323)	0.51	0.39
Age (*N* = 322)	<0.0001	<0.0001
Differentiation (*N* = 187)	<0.0001	0.034
Tumor size (*N* = 279)	0.002	0.0004
Postoperative status (*N* = 309)	<0.0001	<0.0001
Neck metastasis (*N* = 297)	0.74	0.06
Multifocal (*N* = 306)	0.009	0.96
Margin status (*N* = 287)	0.005	0.65
LVI (*N* = 217)	0.0006	0.09
T category (worst of pathology/OR) (*N* = 322)	0.014	0.24
RAI (*N* = 323)	0.62	0.13
RT (*N* = 323)	0.01	0.71
SURGERY (*N* = 290)	0.01	0.074

**Table 3:** Multivariate analysis summary—Cox proportional hazards modeling. (**)

	CSS (*N* = 253 with 26 events)		LRFR (*N* = 263 with 41 events)	
Variable	Hazard ratio (HR) (95% confidence interval (CI))	*P* value	HR (95% CI)	*P* value
Histology	8.7 (3.6–21.0)	<.0001		
Age	3.1 (1.2–8.2)	.02	1.8 (0.9–3.7)	.09
Differentiation (*)				
Tumor size	1.8 (0.7–4.6)	.25	2.1 (1.04–4.1)	.04
Postoperative status	7.1 (2.6–19.6)	.0001	4.6 (2.2–9.8)	<.0001
Neck metastasis			1.4 (0.8–2.7)	.26
Multifocal	0.3 (0.1–0.9)	.026		
Margin status	1.3 (0.5–3.4)	.56		
LVI (*)				
T category (worst of pathology/OR)	1.2 (0.5–3.3)	.68		
RAI	0.5 (0.1–1.9)	.29	0.9 (0.3–2.4)	.76
RT	0.9 (0.3–2.8)	.81	1.4 (0.7–2.9)	.36
SURGERY	0.6 (0.2–1.7)	.36	1.03 (0.5–2.0)	.93

(*) Not considered in the multivariate analysis because of the extent of missing data.

Note. Cox model was stratified by 3 approximately equal time periods: 1958–1971, 1972–1985, and 1986–1998 [11].
